# Brain white matter microstructure alterations in adolescent rhesus monkeys exposed to early life stress: associations with high cortisol during infancy

**DOI:** 10.1186/2045-5380-3-21

**Published:** 2013-12-02

**Authors:** Brittany R Howell, Kai M McCormack, Alison P Grand, Nikki T Sawyer, Xiaodong Zhang, Dario Maestripieri, Xiaoping Hu, Mar M Sanchez

**Affiliations:** 1 Department of Psychiatry & Behavioral Sciences, Emory University, 101 Woodruff Circle, WMB Suite 4000, Atlanta, GA 30322, USA; 2 Yerkes National Primate Research Center, Emory University, 954 Gatewood Road NE, Atlanta, GA 30329, USA; 3 Department of Psychology, Spelman College, 350 Spelman Lane, Box 209, Atlanta, GA 30314, USA; 4 Department of Natural Sciences, Clayton State University, 2000 Clayton State Boulevard, Morrow, GA 30260, USA; 5 Department of Comparative Human Development, University of Chicago, 5730 South Woodlawn Avenue, Chicago, IL 60637, USA; 6 Biomedical Imaging Technology Center, Emory University, 1760 Haygood Drive, Room W232, Atlanta, GA 30322, USA

**Keywords:** Early life stress, Adolescence, Rhesus monkeys, Diffusion tensor imaging

## Abstract

**Background:**

Early adverse experiences, especially those involving disruption of the mother-infant relationship, are detrimental for proper socioemotional development in primates. Humans with histories of childhood maltreatment are at high risk for developing psychopathologies including depression, anxiety, substance abuse, and behavioral disorders. However, the underlying neurodevelopmental alterations are not well understood. Here we used a nonhuman primate animal model of infant maltreatment to study the long-term effects of this early life stress on brain white matter integrity during adolescence, its behavioral correlates, and the relationship with early levels of stress hormones.

**Methods:**

Diffusion tensor imaging and tract based spatial statistics were used to investigate white matter integrity in 9 maltreated and 10 control animals during adolescence. Basal plasma cortisol levels collected at one month of age (when abuse rates were highest) were correlated with white matter integrity in regions with group differences. Total aggression was also measured and correlated with white matter integrity.

**Results:**

We found significant reductions in white matter structural integrity (measured as fractional anisotropy) in the corpus callosum, occipital white matter, external medullary lamina, as well as in the brainstem of adolescent rhesus monkeys that experienced maternal infant maltreatment. In most regions showing fractional anisotropy reductions, opposite effects were detected in radial diffusivity, without changes in axial diffusivity, suggesting that the alterations in tract integrity likely involve reduced myelin. Moreover, in most regions showing reduced white matter integrity, this was associated with elevated plasma cortisol levels early in life, which was significantly higher in maltreated than in control infants. Reduced fractional anisotropy in occipital white matter was also associated with increased social aggression.

**Conclusions:**

These findings highlight the long-term impact of infant maltreatment on brain white matter structural integrity, particularly in tracts involved in visual processing, emotional regulation, and somatosensory and motor integration. They also suggest a relationship between elevations in stress hormones detected in maltreated animals during infancy and long-term brain white matter structural effects.

## Background

Childhood maltreatment is a serious health problem due to both adverse physical and psychopathological outcomes. Adverse outcomes associated with maltreatment include anxiety and mood disorders, substance abuse, conduct disorder, poor impulse control, increased aggression, and other social deficits [1-5]. Infant abuse is not exclusive to humans, but also occurs in wild and captive populations of nonhuman primates, including macaques, chimpanzees, baboons and marmosets [6]. Studies in rhesus monkeys have shown that infant maltreatment also results in socioemotional and stress physiology deficits [7-12] that resemble those seen in maltreated children.

The alterations in behavior and stress physiology exhibited by victims of maltreatment (both human and nonhuman) are hypothesized to be caused by stress-induced differences in brain development, particularly of neural circuits regulating those functions. Studies in humans utilizing MRI have shown alterations in the volumes of specific brain regions including the hippocampus, amygdala, and prefrontal cortex (PFC) in adults with histories of maltreatment [13-18]. Studies investigating alterations in children and adolescents are more inconsistent, and have found more diffuse neural alterations including reductions in temporal, frontal, and parietal cortical volumes as well as decreased corpus callosum (CC) and general cortical white matter (WM) volumes [3,19-22]. This, and additional evidence, supports the view that maturation of brain WM is particularly sensitive to early life stress/adversity [23-27], possibly due to the dramatic developmental changes in myelinated WM, and fiber tracts in general, that occur from childhood through adulthood in both humans [28-35] and nonhuman primates [36-38].

Diffusion tensor imaging (DTI) is a noninvasive, quantitative variation of structural magnetic resonance imaging (MRI) used to measure diffusion of water in the brain. When diffusion is unrestricted, the motion of the water molecules is isotropic, or equal in all directions. However, diffusion is restricted along the axons of myelinated WM tracts, resulting in anisotropic (preferential in one direction) diffusion. The strength of this directional diffusion can be quantified using measures such as fractional anisotropy (FA). Higher FA indicates an increase in the microstructural integrity of the tract, which can be due to several factors, such as increases in myelin thickness, axonal density/diameter, axon neurofilaments/microtubule density, and spread or coherence of fiber orientation in a given voxel [39-42]. Other diffusion properties can be examined to complement investigations of FA because as they provide additional information regarding the mechanisms underlying microstructural differences [43-45]. In particular, radial diffusivity (RD), which quantifies water diffusion perpendicular to the axon and decreases with increased myelination [45-48], and axial diffusivity (AD), which measures diffusivity parallel to the fibers and increases with axonal microorganization, density and caliber, but is not affected by myelin thickness [49,50], can provide valuable information when measured in parallel to FA.

Although the neurobiological mechanisms underlying differences in FA and its functional effects on axonal tract efficiency are not completely understood, there is strong evidence of overall increases in FA (that is tract integrity) in major brain fiber tracts during primate development, although the maturational rates are tract-specific [28,29,33-36]. The role of brain WM tract integrity in behavioral control, particularly during development, is being recognized as an important mechanism underlying behavioral alterations [51] due to its effects on timing and speed of intercellular communication; for example increased tract integrity via increased myelin can increase information transfer via faster conduction speed along the axon [52,53]. Thus, increases in regional FA have been associated with behavioral training and learning [47,48,54-58] and cognitive skills in typically developing children, so that, in general, increased FA has been related to improved behavioral performance [53]. Decreases in FA thought to underlie the poor outcomes related to early stress/adversity have been reported [23,24,26,27,59]. Decreases in FA have also been observed in several psychopathologies including anxiety disorders [60], major depression [61,62], and bipolar disorder [63]. However, increases in FA have also been associated with psychopathology [64-66] and region-specific increases in FA have also been reported in some models of early stress [67,68], suggesting that adverse early experiences affect WM integrity in complex ways, which may depend on factors such as age of exposure, severity of experience/symptoms, and so on.

Prospective studies assessing the impact of childhood maltreatment on brain WM development and the potential mechanisms involved are difficult to perform in children. The goal of the present study was to use DTI to address these questions using a well-established rhesus monkey model of infant maltreatment. In particular we investigated the long-term effects of this adverse early experience on brain WM and behavior during adolescence, and its potential association with stress-induced elevations in cortisol during infancy. Infant maltreatment in this model is comprised of (1) physical abuse, operationalized as violent behaviors exhibited by the mother towards the infant, which reacts with overt signs of distress, and (2) high rates of infant rejection, which is a physically undamaging behavior consisting of pushing the infant away when it solicits contact from the mother, but that also causes infant distress [7,69]. Using this model we have previously reported increased emotional reactivity in maltreated infants and juveniles [7,9,70] and social alterations including delayed independence from the mother and less play during infancy [6,71], as well as increased social aggression during adolescence [72]. Alterations in the hypothalamic-pituitary-adrenal (HPA) stress neuroaxis have also been reported in this maltreatment model, including elevated basal plasma cortisol levels at one month of age, when abuse rates were highest [8,9], which in some cases remain elevated for the first year of life, in parallel with increased stress reactivity [11], and pituitary changes (that is blunted adrenocorticotropic hormone (ACTH) responses to corticotropin-releasing hormone (CRH) administration) that confirmed HPA axis overactivity during infancy [12].

Given all this evidence, in this study we used DTI and tract-based spatial statistics (TBSS) to investigate the long-term effects of infant maltreatment on brain WM tract integrity during adolescence and whether they were related to the increased cortisol levels detected in maltreated animals during their first month of life. WM tract integrity was measured by FA, in parallel with RD and AD measures to aid with the interpretation of the local microstructural mechanisms involved [36,43,45,47,48,54,55,73-75]. In order to assess potential functional correlates of maltreatment-related brain differences, we also examined the associations between brain WM tract integrity and measures of social behavior, in particular aggression, based on reports that it is increased in adolescent maltreated animals as compared to controls [72]. Given the associations reported between early adverse experiences and reduced brain WM tract integrity in children and adolescents, particularly in cortico-limbic tracts and association cortices, including prefrontal-temporal connections [23-25,68,76], we hypothesized that maltreated monkeys would have lower FA in these tracts than control animals. Based on the role of these cortico-limbic tracts in social and emotional regulation, we also hypothesized that lower WM tract integrity would be associated with increased aggression.

## Methods

### Subjects and housing

Nineteen adolescent rhesus monkeys (*Macaca mulatta*) living in four large social groups were used in these studies. Each group consisted of 2 to 3 adult males and 18 to 49 adult females with their sub-adult and juvenile offspring. The groups were housed in outdoor enclosures with access to climate-controlled indoor housing areas located at the Yerkes National Primate Research Center (YNPRC) Field Station, in Lawrenceville, GA, USA. Subjects were given commercially available primate chow (Purina Mills Int., Lab Diets, St. Louis, MO, USA) supplemented with fresh fruit twice daily, and water was available *ad libitum*. All procedures were approved by the Emory University Institutional Animal Care and Use Committee in accordance with the Animal Welfare Act and the US Department of Health and Human Services ‘Guide for Care and Use of Laboratory Animals’.

Of the nineteen subjects in this study, nine experienced maternal maltreatment in the form of physical abuse early in infancy (five females and four males; see operational definition below and in previous publications) [7,12] and the other ten subjects were non-maltreated controls (six females and four males). Following behavioral definitions, observation protocols, and inclusion/exclusion criteria described in detail in previous publications using this same group of nineteen animals [7,9], infant abuse was operationalized as at least three occurrences of the following violent behaviors by the mother towards the infant during the first three months of life: dragging the infant by the tail or leg while running or walking, crushing the infant against the ground with both hands, throwing the infant with one hand while standing or walking, stepping on the infant with one or both feet, sitting on the infant, roughly grooming by forcing the infant to the ground and pulling out the infant’s hair causing distress calls, or carrying the infant with one arm away from the mother’s body thus not allowing the infant to cling [7,12,69]. As mentioned in the Introduction section, all of these abusive behaviors caused distress in the infants, who experienced an average of one and a half events of abuse per hour during their first month of life [7]. Maltreated infants also experienced intense maternal rejection, which involved pushing away the infant when it solicited contact from its mother [7], hence the use of the term maltreatment rather than simply abuse. Subjects in the control and maltreated groups were matched for age, sex, and maternal dominance rank whenever possible so that the two groups did not significantly differ in any of these variables.

### HPA axis basal activity: cortisol in infancy

Basal blood samples were collected at sunrise from all subjects when they were one month old, coincident with the highest rates of abuse [7], following published protocols [9,12,77]. Plasma concentrations of cortisol were measured in duplicate 10 μL aliquots by radioimmunoassay using commercially available kits (Diagnostic Systems Laboratories, DSL, Webster, TX, USA). Although we have already reported elsewhere that maltreated animals have greater plasma cortisol levels at one month of age than controls [8,9], these cortisol concentrations were used in the current study to examine their correlations with brain structural measures during adolescence (see details below).

### Behavioral data collection during adolescence

Social behavior was collected around four years of age (close to 48 months) from observation towers located in the corners above each subjects’ social home compound. Data was collected between 7 and 11 am, when animals are most active, using an established rhesus ethogram [78] with modifications [70]. This behavioral data was collected by three trained observers using binoculars and handheld computers (Palm IIIxe, Palm Inc., Sunnyvale, CA, USA) programmed to collect durations, frequencies, and sequences of behavior [79]. Inter-observer reliability was calculated prior to real time collection of behavior, by having each observer watch and record behavior from videos until percent agreement reached at least 90% and Cohen’s Kappa was greater than 0.8.

Frequency of aggressive behaviors was measured using five hours of focal observations in each animal (five separate sessions, one hour each). Behaviors categorized as aggression included biting, grabbing, pinning, threatening and chasing of others in the group. A composite score of the frequencies of all of these behaviors was used to calculate the frequency of total aggression used in the analysis as rates per hour. Although increased social aggression has been reported in these maltreated animals as a separate and more extensive study of affiliative and agonistic behavior in these animals [72], total aggression rates per hour (average of contact and non-contact aggression) data were used in the current study to examine its associations with brain structural measures collected at similar ages (see details below).

#### In vivo neuroimaging

##### T1-Weighted MRI acquisition and template construction

**Figure 1:**
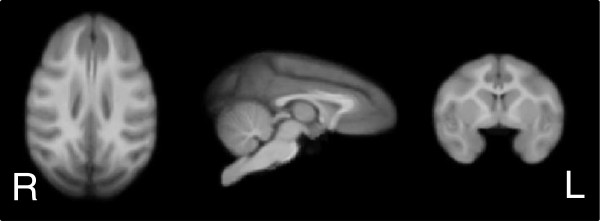
**Study specific template of four and a half-year-old rhesus monkeys produced using iterative affine registrations and averaging as previously described **[78]**.**

Imaging data was acquired during adolescence, beginning at four years of age (range: 48 to 55 months; mean ± SEM scan ages were: maltreated animals = 51.99 ± 0.6 months, controls = 51.98 ± 0.57 months). The scanning age was not different between control and maltreated animals, as described in the Results section. Structural (T1-weighted MRI) images were acquired during the same scanning session as the DTI scans on a 3 T Siemens Trio scanner (Siemens Medical Solutions USA, Inc., Malvern, PA, USA) at the YNPRC Imaging Center using a transmit and receive volume coil (Siemens CP Extremity Coil, Siemens Medical Solutions USA, Inc., Malvern, PA, USA) and a magnetization prepared rapid gradient echo (MPRAGE) sequence with the following parameters: TI/TR/TE = 950/3000/3.3 ms; flip angle = 8 degree; total scan time = 38 min; FOV = 116 mm × 116 mm × 96 mm, with a 192 × 192 × 160 matrix and 4 averages; voxel size: 0.6 × 0.6 × 0.6 mm^3^. A T1 template was constructed from these scans using the methods described for rhesus monkeys by McLaren and colleagues [80]. Briefly, first a single subject was affinely registered to the rhesus monkey atlas developed at the University of Wisconsin [80] resulting in a single subject in Wisconsin 112RM-SL rhesus atlas space (the target image), which is in the brain coordinate space of the Saleem-Logothetis rhesus stereotaxic atlas [81]. Each of the other subjects was then affinely registered to the target image and all of these images (now in atlas space) were averaged. This first-run template was then used as the target for a second round of affine registrations and averages resulting in a 0.5 × 0.5 × 0.5 mm^3^, study-specific average T1 image that was used as a template for the analyses described below (Figure 1).

##### DTI data acquisition, preprocessing, and analysis

Whole brain DTI data was acquired using a dual spin-echo, segmented (multi-shot) diffusion-weighted echo-planar imaging (EPI) sequence with the acquisition parameters: TR/TE = 6,000/90 ms, 4 shots, b: 0, 1,000 s/mm^2^, FOV = 96 mm × 96 mm, slice thickness = 1.5 mm with zero gap, voxel size = 1.5 × 1.5 × 1.5 mm^3^, 30 slices, 64 × 64 matrix, 30 directions, and 4 averages.

**Figure 2:**
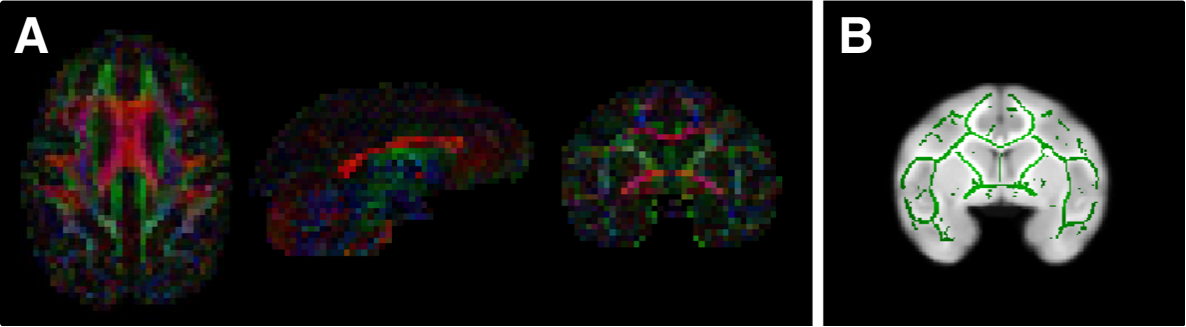
**Representative diffusion tensor imaging (DTI) in four and a half-year-old rhesus monkeys. (A)** Fractional anisotropy (FA) color map. Red represents left-right oriented fibers, blue represents dorsal-ventral oriented fibers, and green represents anterior-posterior oriented fibers. **(B)** Mean FA skeleton displayed on study specific template.

The DTI data was corrected for B0 inhomogeneity-induced distortion [82] and eddy current effects [83] using the FSL software (FMRIB Center, University of Oxford, Oxford, UK) [84]. FA, RD, and AD were calculated using the diffusion analysis tools in FSL [84] (Figure 2). The TBSS tool in FSL [85] was used as a voxel-wise approach to identify the centers of all major WM tracts present in all subjects, therefore reducing the number of multiple comparisons. TBSS first nonlinearly registers each subject’s FA image to the template image (the study specific T1 template produced as described above, resulting in a final image resolution of 0.5 × 0.5 × 0.5 mm^3^). These images were then averaged to create a mean FA image from which a mean FA skeleton was created (see Figure 2B) using a user-defined FA threshold. The threshold applied in the current study was 0.2 to avoid inclusion of small peripheral white matter, and is a common threshold used for this type of analysis [85], and has been previously used by our group in studies in rhesus monkeys [68]. To reduce the effects of misregistration on the FA values contained within each subjects’ skeletonized data, the TBSS software searches the voxels surrounding the mean FA skeleton in each subjects’ registered FA image to assign the highest local FA value for each subject to the skeleton (for complete description see [85]). This ensures that despite the fact that the mean FA skeleton does not exactly cover the same anatomical regions in all subjects, the FA values contained in each subject’s skeletonized data do represent the centers of the major WM tracts of each individual subject. These FSL diffusion analysis tools have been previously applied with success to rhesus brain DTI data by our group [68,86] and others [87-90].

### Statistical analyses

#### Statistical analysis of FA, RD, and AD data

A voxel-wise two-group *t*-test was performed on the skeletonized FA data using the Randomise tool in FSL [84] to determine regions with significant differences between the maltreated and control groups. Results were considered significant at *P*-value less than 0.005 (uncorrected, but using a minimum cluster volume of 10 μL, approximately 4.5 significant contiguous voxels in native diffusion space) due to the relatively low spatial resolution. Results (significant clusters of > 4.5 contiguous significant voxels in native diffusion space) were displayed in the T1 study-specific template described above, registered to the Wisconsin 112RM-SL rhesus atlas [80,91], which is in the coordinate space of the Saleem-Logothetis rhesus brain stereotaxic atlas [81].

Binary masks were created for the clusters showing group FA differences. The mean RD and AD values were calculated within these regions, following previously published approaches [54,68,84]. A two-group *t*-test was performed on these values to determine the effects of infant maltreatment on RD and AD in those clusters with significant FA differences to aid in identifying the underlying microstructural mechanisms of the differences in tract integrity (significance level was set at *P* < 0.05). The mean FA calculated for each cluster was also used to examine its correlations with infant cortisol and adolescence aggression data using Pearson correlation (see details below).

#### Correlations between FA and biobehavioral measures (cortisol and aggression)

Because we were interested in examining the associations between infant cortisol levels and long-term alterations in tract integrity (that is, FA) detected as a consequence of this early adverse experience, as well as functional correlates of FA group differences during adolescence, we performed Pearson product moment correlation analyses restricted to those regions (clusters) where group differences in FA were detected above. Control and maltreated groups were included together in the correlation analyses between FA and basal plasma cortisol levels at one month of age and aggression during adolescence. Statistical significance level was set at *P* < 0.05.

## Results

### Group differences in FA

**Figure 3:**
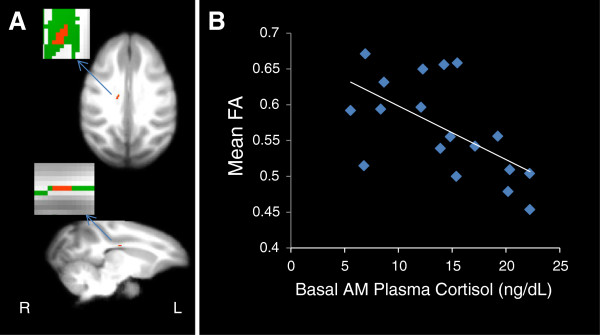
**Maltreated animals have reduced fractional anisotropy (FA) in the corpus callosum. (A)** Cluster of voxels (red) in right corpus callosum represents the region where maltreated animals had significantly lower FA than controls (*p* < 0.005, uncorrected, ≥ 10 μL volume). In insets, green represents the mean FA skeleton. **(B)** FA in the corpus callosum is negatively correlated with basal cortisol at one month of age (r = −0.512, *P* = 0.025).

**Figure 4:**
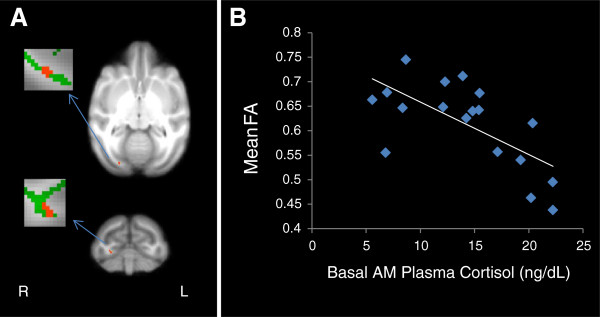
**Maltreated animals have reduced fractional anisotropy (FA) in right occipital white matter (WM). (A)** Cluster of voxels (red) in right occipital WM where maltreated animals had significantly lower FA than controls (*P* < 0.005, uncorrected, ≥ 10 μL volume). In insets, green represents the mean FA skeleton. **(B)** FA in right occipital WM is negatively correlated with basal cortisol at one month of age (r = −0.561, *P* = 0.012).

**Figure 5:**
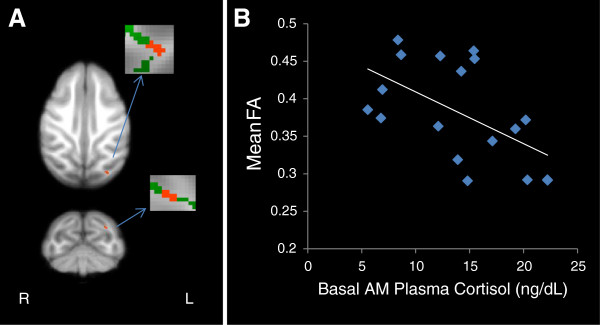
**Maltreated animals have reduced fractional anisotropy (FA) in left occipital white matter (WM) (left occipital cluster 1). (A)** Cluster of voxels (red) in left occipital WM where maltreated animals had significantly lower FA than controls (*P* < 0.005, uncorrected, ≥ 10 μL volume). In insets, green represents the mean FA skeleton. **(B)** FA in left occipital WM is negatively correlated with basal cortisol at one month of age (r = −0.483, *P* = 0.036).

**Figure 6:**
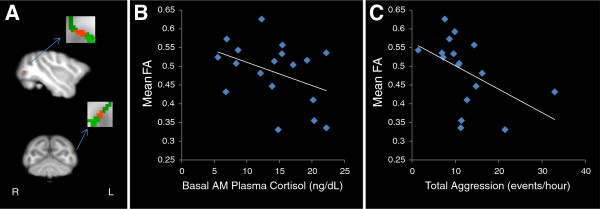
**Maltreated animals have reduced fractional anisotropy (FA) in left occipital white matter (WM) (left occipital cluster 2). (A)** Cluster of voxels (red) in left occipital WM where maltreated animals had significantly lower FA than controls (*P* < 0.005, uncorrected, ≥ 10 μL volume). In insets, green represents the mean FA skeleton. **(B)** FA in left occipital WM is negatively correlated with basal cortisol at one month of age (r = −0.479, *P* = 0.038). **(C)** FA in left occipital WM is negatively correlated with total aggression in adolescence (r = −0.465, *P* = 0.045).

**Figure 7:**
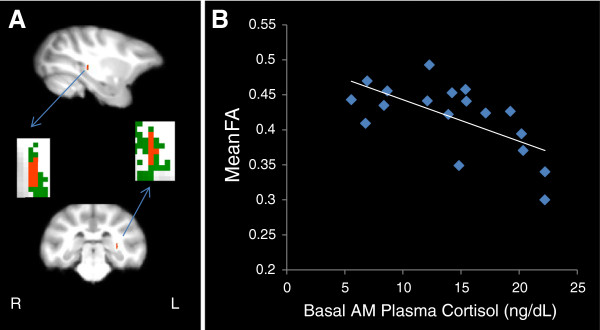
**Maltreated animals have reduced fractional anisotropy (FA) in white matter (WM) dorsal to the hippocampus and lateral to the pulvinar nucleus. (A)** Cluster of voxels (red) where maltreated subjects had significantly lower FA than controls (*P* < 0.005, uncorrected, ≥ 10 μL) seems to correspond to the external medullary lamina (EML). In insets, green represents the mean FA skeleton. **(B)** FA in the EML is negatively correlated with basal cortisol at one month of age (r = −0.637, *P* = 0.003).

**Figure 8:**
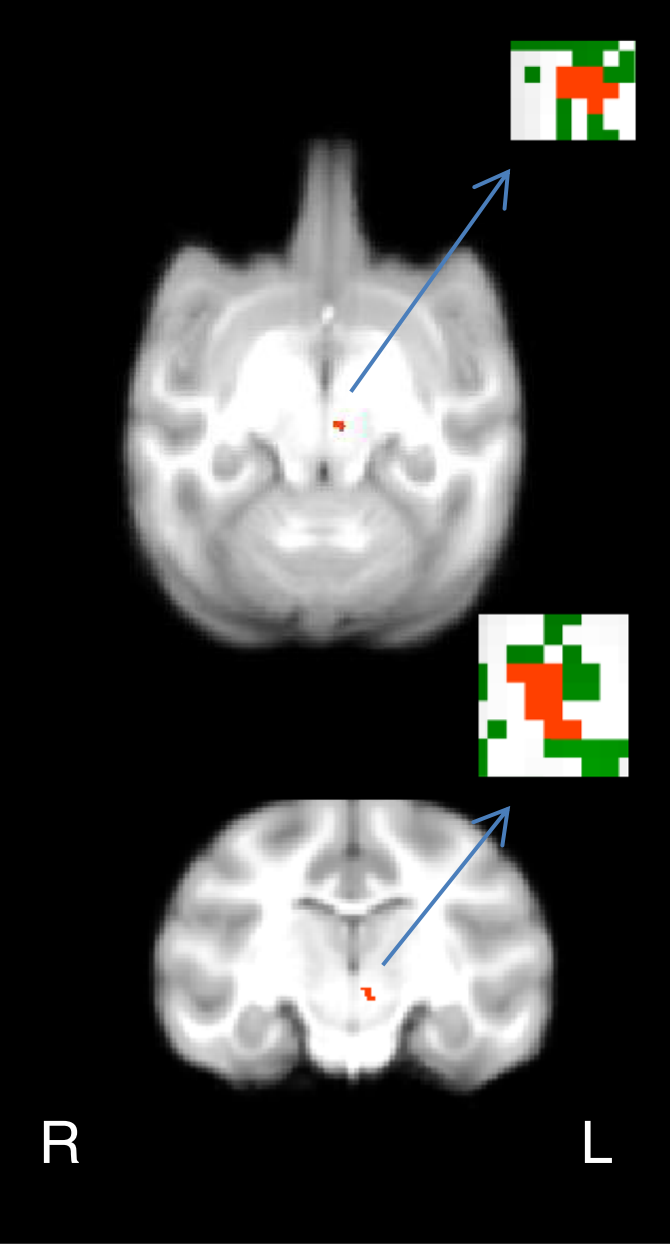
**Maltreated animals have reduced fractional anisotropy (FA) in brainstem white matter (WM).** The cluster of voxels (red) in left brainstem WM where maltreated animals had significantly lower FA than controls (*P* < 0.005, uncorrected, ≥ 10 μL) could correspond to the central tegmental tract (CTT). In insets, green represents the mean FA skeleton.

No differences in scanning age were detected between maltreated and control animals (*P* = 0.99; Student *t*-test). Significantly lower FA (*P* < 0.005, uncorrected, cluster volume ≥10 μL) was observed in maltreated animals, in comparison to controls, in six clusters: (1) one in WM located in the lateral portion of the medial midbody of the CC [92] (Figure 3A); (2) one in right occipital WM (Figure 4A); (3) two clusters in left occipital WM (Figures 5A and 6A), which, along with the cluster located in right occipital WM could include the inferior longitudinal fasciculus (ILF) or possibly short intra-occipital fiber systems; (4) one in the WM dorsal to the left hippocampus and lateral to the pulvinar nucleus, which could correspond to the external medullary lamina (EML) (Figure 7A); and (5) one in the brainstem, in a location that matches the position of central tegmental tract (CTT) (Figure 8). No regions were found in which the maltreated animals had significantly higher FA than controls.

### Group differences in RD and AD in regions with significant FA effects

The mean RD and AD values were calculated for each of the clusters with significant group differences in FA. In all clusters, except for the brainstem cluster, decreased FA was accompanied by an increase in RD, suggesting that the difference in FA was due to decreased myelin [42,45-48,93,94]. No differences in AD were observed in any of the clusters with FA effects.

### Correlations of biobehavioral measures with FA

As mentioned above, our group has previously reported elevated plasma cortisol levels during infancy (at one month of age) [9], as well as increased aggression towards group mates during adolescence (at approximately four years of age) in the maltreated animals, which are the focus of this study, in comparison to controls [8,9,72]. Therefore, only results of the correlations between FA and these biobehavioral measures are presented here. The mean FA value of each cluster in which significant group differences in this measure were found was correlated with infant basal cortisol and frequency of aggressive behaviors during adolescence. Neither RD nor AD values were included in the correlation analyses because they are components of, and thus correlated with, FA.

Negative correlations between FA and infant cortisol were found in all clusters except for the one in the brainstem (see Table 1) (Figures 3B, 4B, 5B, 6B, and 7B). A negative correlation (Table 1) between aggression and FA was also found in one of the clusters in left occipital WM (Figure 6C), but in none of the other clusters examined.

**Table 1:** Correlations of fractional anisotropy (FA), one month cortisol, and total aggression in adolescence

	**Month 1 cortisol**		**Total aggression**	
Cluster location	*r*	*P*	*r*	*P*
Corpus callosum	−0.512	0.025^a^	−0.31	0.181
Right occipital	−0.561	0.012^a^	−0.113	0.645
Left occipital 1	−0.483	0.036^a^	−0.281	0.244
Left occipital 2	−0.479	0.038^a^	−0.465	0.045^a^
EML	−0.637	0.003^a^	−0.254	0.293
Brainstem	−0.315	0.189	−0.317	0.118

Pearson correlation analysis was used. ^a^*P* < 0.05 was considered significant.

## Discussion

The main goal of this study was to examine the long-term consequences of infant maltreatment on brain WM tracts of adolescent rhesus monkeys and to determine whether they were related to the elevated cortisol levels reported in these maltreated animals during infancy [8,9]. We also examined whether alterations in brain WM microstructure were related to the increased aggressive behavior previously reported in the maltreated animals during adolescence [72]. To do this we used measures of microstructural integrity, specifically FA, RD, and AD, calculated from DTI scans. We chose this technique because of its sensitivity to changes in WM microstructure, such as myelin thickness and axon/microtubule density [39-41]. These are neuronal characteristics that can affect the timing and speed of intercellular communication [52,53], and can therefore affect behavior [51]. FA increases significantly in brain WM tracts throughout primate development, and is accompanied by decreases in RD and few changes in AD [28,29,34-36]. These developmental changes in measures of axon microstructure suggest a global increase in tract integrity mainly due to increases in myelin from childhood to adulthood. Brain region-specific increases in FA are also observed after training on visuo-motor tasks [58] and with acquirement of new cognitive skills, such as reading and math, in parallel to decreases in RD, but no changes in AD [47,48,53,56]. This suggests that these experience-related and region-specific increases in FA are due to increases in myelin and underlie behavioral and cognitive improvements. In contrast, reduced FA, associated in most regions with elevated cortisol during infancy and with increased concurrent aggression in one of the clusters, was detected here in adolescent rhesus monkeys that experienced infant maltreatment. Our findings are consistent with previous reports in human individuals that experienced childhood maltreatment [25,27] or other forms of early life stress [23,24] and in other nonhuman primate models of adversity [26], as well as in several mood and anxiety disorders [66,95], with significant overlap with the regions affected in the current study.

To our knowledge, this is the first DTI study to examine the long-term effects of infant maltreatment on brain WM tract integrity in a nonhuman primate model. It is also the first to examine the associations of brain structural alterations with infant cortisol elevations and concurrent social behavior. Our findings show alterations in brain WM tract integrity measured using DTI in adolescent rhesus monkeys with histories of infant maltreatment. Decreased WM integrity (that is, FA) was found in maltreated subjects in the CC, occipital WM, EML, and brainstem, in comparison to controls. These regional FA decreases were paralleled by increases in RD, but no changes in AD, suggesting that the alterations in tract microstructural integrity in these brain regions were likely due to reduced myelin [42,45-48,93,94]. An exception was the brainstem cluster, where no RD differences were found between groups. Basal plasma cortisol levels measured when the individuals were one month old, when abuse rates were highest [7], were negatively correlated with FA in all regions except for the brainstem cluster. This suggests that maltreatment at that early age caused stress-induced elevations in cortisol that could have potentially contributed to the long-term brain WM alterations reported. However, future studies are needed to examine causality in this relationship.

One of the clusters with lower FA in maltreated animals than controls was located in the lateral aspect of the medial midbody of the CC [92]. The CC is the largest WM tract in the brain conveying interhemispheric fibers important for integration of information between cortical regions in both hemispheres [96]. Because these fibers are some of the last to myelinate [31,32,36,37], finding alterations in the CC is consistent with the view that areas undergoing active myelination or other protracted developmental processes are especially vulnerable to environmental experience [97,98]. Alterations in the CC have also been reported in several studies of maltreated children, with reduced CC volume reported in maltreated children [99,100], a difference that appears to be related to a failure to show the typical age-related increase in volume [101]. Reduced CC size has also been reported in adults with histories of childhood maltreatment [102], suggesting that these CC alterations are persistent. Decreased FA in the CC of maltreated children [103] and adults who have experienced various forms of early life stress [104] has also been reported. The findings of the current study are also consistent with findings of reduced CC size in other nonhuman primate models of adverse early experience [92]. Our findings of reduced WM integrity in the CC medial midbody region, which carries some prefrontal but mostly frontal motor and somatosensory fibers [105], could result in group differences in integration of motor and somatosensory information. The reduced interhemispheric integration reported here and in human studies of childhood maltreatment could contribute to behavioral alterations and psychopathology, an idea supported by similar CC alterations reported in anxiety and mood disorders [106].

The location of the three clusters identified in occipital WM suggest that the tracts affected could include short intra-occipital fiber systems (possibly part of the forceps major, an interhemispheric tract that connects occipital cortices in both hemispheres), and/or the caudal portion of the ILF, a long cortico-cortical association tract that courses through occipital, parietal, and temporal cortices [96]. However, this can’t be corroborated without running additional tractography analyses. Interestingly, reduced FA has been reported in the forceps major of adolescents with histories of child maltreatment [27] and in the caudal portion of the ILF in adolescents that witnessed domestic violence as children [107]. The ILF is part of the ventral visual pathway which is important for object identification [108], face processing [109], and emotional memory [110,111]. Along these lines, alterations in WM microstructure of the ILF have been observed in several mood and anxiety disorders. For example, decreased FA in the ILF at the level of the occipital lobe has also been found in patients with depression [112,113] and bipolar disorder [114,115]. Thus, it is possible that decreases in microstructural integrity of occipital WM, likely involving the ILF, could affect visual and face processing, as well as emotion/mood processes.

The negative correlation of FA with aggressive behavior detected in occipital WM is difficult to explain. Most neuroimaging studies involving neural substrates of aggression implicate structural and/or functional abnormalities in frontal brain circuits [116,117],although many of these studies have been done in patients with schizophrenia. Decreased FA in the anterior commissure (AC) has also been reported in violent youth with bipolar disorder, and FA in the AC was negatively correlated with aggression [118]. However, this study was done in a clinical population making it difficult to integrate with the findings reported here. Increased occipital WM volume has been reported in adult violent offenders [119], but to our knowledge no other occipital alterations have been associated with aggression. Interestingly, a recent study comparing neural systems supporting social cognition in chimpanzees and bonobos reported that chimpanzees (known to be more aggressive than bonobos) had higher FA in occipital WM and bigger occipital GM volumes than bonobos [120], suggesting a potential association between aggression and FA in occipital WM in these species. The discrepancy of the directionality of the correlation with our findings could be explained by factors such as species-specific differences in neural substrates of aggression, or age at measurement. Given the paucity of research on the neural substrates of aggression, particularly in children, the interpretation of our findings is difficult. The visual cortices located near the cluster in which FA and aggression were correlated are part of attentional networks [121], and thus alterations in these circuits could reflect more general alterations in attention that might be better reflected by other behaviors not measured in the current study. It also has to be noted that our small sample size is a limitation for these studies, which might have been underpowered to detect other significant associations.

The WM cluster located lateral to the pulvinar thalamic nucleus and dorsal to the hippocampus seems to be the EML based on rhesus brain atlases [81]. The EML contains both thalamo-cortical and cortico-thalamic fibers connecting the thalamus with parietal, temporal, occipital, cingulate, motor and PFC [96]. Although without performing tractography it is difficult to precisely identify the specific thalamic nuclei and cortical regions connected by the affected tracts, based on the rostro-caudal location of this cluster the fibers affected likely connect the thalamus with occipital or temporal cortices [96]. Interestingly, thalamo-cortical systems modulate amygdala activity, and are involved in the perception of fear [122]. Cortico-thalamic circuits are implicated in the pathogenesis of mood disorders [123]. Thus, our findings of reduced structural integrity in EML suggest potential alterations in cortico-thalamic and thalamo-cortical circuits that could contribute to deficits in emotional regulation reported above in maltreated animals.

The brainstem cluster where FA was lower in maltreated animals than controls was difficult to identify anatomically due to the low MRI contrast in this region. However, as described above, its location matches the position of the CTT [124]. The CTT is a pathway containing descending fibers from midbrain nuclei that project to the olivary complex, as well as ascending fibers originating in the pontine and medullary reticular formation that project to the thalamus [125]. These are brainstem pathways that carry and coordinate somatosensory and somatomotor information. MRI studies report lesions in the CTT in neurodegenerative and neurodevelopmental disorders, linked with motor and cognitive deficits [126]. This was the only region where group differences in FA (lower in maltreated subjects than in controls) were not related to the increased levels of cortisol during infancy in the maltreated animals, suggesting that the effects of maltreatment on this WM could be associated with other aspects of the early experience.

There are limitations to the DTI method as applied here. Most are due to the low spatial resolution of the diffusion data acquired in the relatively small rhesus brain. At this resolution partial voluming effects can make interpreting or finding results difficult. The TBSS analysis applied here addresses this limitation by using only voxels from the centers of large WM tracts in individual subjects. Partial voluming can also make registration difficult, which is another reason why we used the nonlinear registration built into the TBSS processing pipeline to perform our voxelwise analyses. The low angular resolution (that is, the small number of directions acquired for the DTI data), especially when combined with the low spatial resolution of our data, also makes accurate probabilistic tractography difficult, which is why it was not performed in these studies. Tractography would be helpful in future studies to determine the exact tracts affected in the clusters with group differences, although it would not help in determining the directionality of the affected fibers.

The correlations between infant cortisol and WM integrity found in the current study suggest that early life stress has long-term effects on brain WM in regions previously reported as vulnerable to childhood maltreatment in humans, and that are also altered in anxiety and mood disorders. One possible mechanism could be through the effects of elevated levels of glucocorticoids (GCs), in this case cortisol, on the development of WM [127]. Oligodendrocytes that form the myelin sheath express both intracellular glucocorticoid and mineralocorticoid receptors [128], and recent evidence suggests that GCs suppress proliferation of oligodendrocyte precursor cells in GM and WM [129]. Developmental studies also provide evidence that GCs modulate oligodendrocyte differentiation and myelogenesis via regulation of key oligodendroglial proteins such as myelin basic protein (MBP) [130], and that the effects of synthetic GCs differ as function of gestational age, with decreases in MBP immunoreactivity and numbers of oligodendrocytes associated with younger ages of GC exposure [131]. Taken together, these studies suggest that myelination is sensitive to GCs during development, making it possible for early life stress, via elevated cortisol levels, to affect brain WM development. The associations detected in our studies between decreased FA and basal cortisol levels at one month are consistent with this possibility, although the causality of this relationship needs to be tested in future studies. Due to the strong role of brain WM in behavioral control, for example, [132], GC-induced alterations in brain WM development could potentially lead to the alterations reported in maltreated monkeys, including increased aggression. Our findings also open new questions and hypotheses that need to be empirically tested. Does maltreatment lead to altered function of the affected circuits? When do these differences emerge and how they unfold? Prospective, longitudinal studies beginning at birth are necessary to address these important developmental questions in the context of maltreatment to determine the most beneficial timing and type of potential treatments, as well as intervention and prevention strategies.

## Conclusions

The results of the current study suggest that early life stress in the form of infant maltreatment has long-term effects on brain WM in regions that are vulnerable to childhood maltreatment in humans, and that are also altered in anxiety and mood disorders. These findings highlight the long-term impact of infant maltreatment on brain white matter structural integrity, particularly in tracts involved in visual processing, emotional regulation, and somatosensory and motor integration. They also suggest a relationship between long-term brain white matter structural effects and elevations in stress hormones detected in maltreated animals during infancy, as well as aggression during adolescence.

## Abbreviations

AC: Anterior commissure; ACTH: Adrenocorticotropic hormone; AD: Axial diffusivity; CC: Corpus callosum; CCT: Entral tegmental tract; CRH: Corticotropin releasing hormone; DTI: Diffusion tensor imaging; EML: External medullary lamina; EPI: Echo planar imaging; FA: Fractional anisotropy; FMRIB: Oxford Centre for Functional MRI of the Brain; FSL: FMRIB Software Library; GC: Glucocorticoid; GM: Gray matter; HPA: Hypothalamic-pituitary-adrenal axis; ILF: Inferior longitudinal fasciculus; MPRAGE: Magnetization prepared rapid gradient echo; MRI: Magnetic resonance imaging; PFC: Prefrontal cortex; RD: Radial diffusivity; TBSS: Tract based spatial statistics; WM: White matter; YNPRC: Yerkes National Primate Research Center.

## Competing interests

The authors declare that they have no competing interests.

## Authors’ contributions

BRH carried out the processing and analyses of the DTI data, and drafted the manuscript. KM, APG, and NTS developed the experimental design of the aggression studies and collected and analyzed the data. MMS developed the experimental design of the overall studies, and some of the grant proposals that funded them. She also participated in the collection and analysis of the cortisol and DTI data and in the writing of the manuscript. DM participated in the experimental design of some of the studies and developed one of the grant proposals that funded them. XH and XZ developed the DTI scanning sequences used here for the rhesus monkey brain. All authors read and approved the final manuscript.
